# Molecular Analysis of Disease-Responsive Genes Revealing the Resistance Potential Against Fusarium Wilt (*Fusarium udum* Butler) Dependent on Genotype Variability in the Leguminous Crop Pigeonpea

**DOI:** 10.3389/fgene.2020.00862

**Published:** 2020-08-20

**Authors:** Koushik Biswas, Avijit Tarafdar, Roshan Kumar, Nirjara Singhvi, Parthadeb Ghosh, Mamta Sharma, Sunil Pabbi, Pratyoosh Shukla

**Affiliations:** ^1^Department of Biotechnology, Visva Bharati University, Santiniketan, India; ^2^Legumes Pathology, International Crops Research Institute for the Semi-Arid Tropics, Hyderabad, India; ^3^Department of Human Genetics and Molecular Medicine, Central University of Punjab, Bathinda, India; ^4^Department of Zoology, University of Delhi, New Delhi, India; ^5^Department of Botany, University of Kalyani, Kalyani, India; ^6^Centre for Conservation and Utilisation of Blue Green Algae, Division of Microbiology, Indian Agricultural Research Institute, New Delhi, India; ^7^Enzyme Technology and Protein Bioinformatics Laboratory, Department of Microbiology, Maharshi Dayanand University, Rohtak, India

**Keywords:** disease incidence, *Fusarium udum*, gene expression, innate resistance, pathogenesis, pigeonpea

## Abstract

Fusarium wilt (FW), caused by *Fusarium udum* Butler (FU), is among the challenging factors in the production of pigeonpea. Therefore, exploring a superior pigeonpea genotype from landraces or local cultivars through the selection of innate resistance to FW using different biological and molecular approaches, and validating its resistance response, could be an alternative to sustainable crop improvement. Five distinct pigeonpea genotypes, with resistant (ICP2894) and susceptible (ICP2376) controls, were selected on the basis of the incidence percentage of FW, from three different states of India. Among them, the cultivar Richa, which displayed low incidence of FW (10.0%) during the genotype evaluation, was further examined for its innate resistance to FW. Molecular characterization of antioxidant (AO) enzyme [*APX* and *SOD*] and pathogenesis-related (PR) protein [*CHS* and β*-1, 3-glucanase*] families were performed. The obtained results of reverse transcription-polymerase chain reaction-based expression study and *in silico* analysis showed a higher level of induction of PR and AO genes, and the strong interaction of their putative proteins with fungal cellobiohydrolase-c protein established their antifungal activity, conferring early plant defense responses to FU in Richa. Our study demonstrated a strong and combinatorial approach involving biological assay, molecular experiments, and *in silico* analysis to identify a superior pigeonpea genotype that was resistant to FW across a major biogeographic region.

## Introduction

The global population is continuously increasing and is projected to increase further by 3.5 billion by the end of this century, to an estimated number of 11.2 billion (Max, 2019), resulting in increased demand for food grains. With long-term planning for the sustainable genetic improvement of major staple crops such as rice, wheat, and maize, it is necessary to simultaneously make a breakthrough in the uninterrupted production of proteinaceous foods to maintain a balance for reducing global hunger and malnutrition. Legumes, preferably pigeonpea, can be considered to be a good alternative to easily available protein resources and offers a considerable quantity of food proteins in the Indian and African subcontinent, with lesser cultivation care and low inputs. Pigeonpea belongs to the family *Fabaceae* and contributes by fulfilling the protein demand of approximately 20% of the global population. It also serves as a major source of vitamins. In India, pigeonpea stands second after chickpea as the most important food legume crop (Allen and Lenné, 1998). However, recently, a major restraint in pigeonpea production has been caused by biotic stresses, among which, Fusarium wilt (FW) is considered to be the most devastating disease, followed by sterility mosaic disease and Phytophthora blight. The former accounts for 30–100% losses in the yield of pigeonpea genotypes that are vulnerable to this disease (Pande et al., 2011).

Assessment of the effect of any disease on the yield of a crop, with the help of a complete survey, is a pre-requisite for preparing a rational disease management program. The system biology approach encompasses that the impact of biotic stress, especially pathogen attack in combination with other variable abiotic stress factors, leads to the molecular fine-tuning of the degree of resistance, tolerance, or susceptibility of a specific host plant (Dangi et al., 2018). On the other hand, sources of resistance identified in one region do not necessarily confer the same degree of resistance in other regions, thereby indicating pathogenic variability in the fungus (Kumar and Upadhyay, 2014). Although several new sources of resistance have been reported against FW, there are still many possibilities of obtaining improved and potential germplasms or indigenous genotypes at the regional or rural level, by searching, collecting, and evaluating such genotypes for better resistance, using a standard inoculation method (Pande et al., 2012). The present study on evaluating the germplasm against FW has also revealed the importance of studying the epidemiology of wilt disease. The genetics of FW resistance and/or tolerance in pigeonpea is yet to be fully understood, and many more genes, from a single dominant gene to two complementary genes, are supposed to be involved, and there might even be involvement of multiple genetic factors (Okiror, 2002). The in-depth knowledge of the genetics of resistance mechanisms in host plants and genomic insights of wilt-causing pathogen *Fusarium udum* (FU) is equally crucial for the development of effective strategies for the efficient transfer and stable function of such resistant genes into disease-susceptible cultivars. A number of resistant sources of germplasm against FW were identified by screening various pigeonpea genotypes over the last two decades at the national and international levels. Some of these promising genotypes have been effectively used in crop improvement programs (Joshi, 2001; Joshi et al., 2000; Okiror, 2002; Mishra et al., 2003; Madhukeshwara et al., 2004; Pande et al., 2012).

The fabrication of defense molecules such as phytoalexins, phenylpropanoids, and pathogenesis-related (PR) proteins, such as chalcone synthases (CHSs), chitinases, and β-1,3-glucanases play crucial roles because of their direct activity on the structural components of fungi and insects. Besides these, a large number of cellular enzymes related to biotic stresses, such as superoxide dismutase (SOD), catalases, and peroxidases are overexpressed to get rid of the invading pathogens (Sela-Buurlage et al., 1993). Two PR protein-associated enzymes, namely chitinase and β-1,3-glucanase, were successfully extracted and purified by gel filtration from the fungal pathogen-induced chickpea plants, and their higher activity was validated by inhibiting the growth of *Fusarium oxysporum* f. sp. *ciceris* (Foc) and other phytopathogenic fungi (Saikia et al., 2005). The response of the defense network in chickpea plants against the strain was also established by highlighting the studies on confocal microscopy to trace the pathogen invasion, various enzymatic assays to detect structural changes in the plant cellular membrane, and validation of the defense responsive transcripts by qRT-PCR approach (Gupta et al., 2013). A microarray-based genomewide transcriptional study on tomato plants infected with *F. oxysporum* f. sp. *lycopersici* (*Fol*) and tomato mosaic virus has revealed a strong homeostatic defense response, wherein chitinase and other PR gene families displayed high-level positive regulation in terms of fold changes (Andolfo et al., 2014). Molecular cloning and characterization of the β-1, 3-glucanase gene in wheat inoculated with the stripe rust pathogen has demonstrated a high level of expression of this gene using qPCR analysis (Liu et al., 2010).

The existence of different variants/races of FU has already been reported from different pigeonpea growing regions of India. Considering the economic importance of pigeonpea and the severity of FW disease, the present study was conducted to explore the existence of host resistance in pigeonpea genotypes with high tolerance to wilt. The main reason and the core focus of our study was on discovering new pigeonpea genotypes/landraces resistant to FW, popularly grown locally but not widely in India, and to study the genetic background behind their resistance reaction to FW. A preliminary screening of pigeonpea genotypes collected from different states of India, based on resistance against a highly virulent FU strain, was performed to identify resistant/tolerant genotypes. Attempts were made to identify the defense-related genes in resistant/tolerant genotypes by applying molecular and *in silico* approaches, and thereby investigate their expression pattern using quantitative PCR (qPCR) during pathogen infection. The present report describes a detailed biological and molecular study to investigate the genetics of tolerance mechanism of some well-known defense-responsive and antioxidant (AO) genes in pigeonpea against FW.

## Materials and Methods

### Survey of the Pigeonpea-Growing Areas in India

Surveys were conducted at 25 villages of nine districts from major pigeonpea-growing regions in five different states of India viz. Andhra Pradesh (AP), Madhya Pradesh (MP), Tamil Nadu (TN), Telangana (TS), and West Bengal (WB). The prime objective of this study was to discover new pigeonpea genotypes/landraces grown locally and resistant to FW from the areas where lack of cultivation practices of popular hybrids by the local farmers (Table 1). Therefore, the surveyed areas were mostly selected based on regions where the farmers rely on local cultivars. So, there might be a chance of the presence of natural hybrid, which was adopted by the farmers since long back. The surveys were carried out using a random sampling method. Epidemiological study of FW in pigeonpea was performed using the formula of percent disease incidence (DI%), where DI% = number of plants infected by wilt disease × 100/Total number of plants observed. The DI% was individually calculated for each plot using the above formula. The DI% of three plots of a single field were summed to determine the mean DI%. The average values representing the overall DI% for each village under survey were arranged in a tabular format (Table 1). To understand the epidemiological intensity of the disease in each village of the respective surveyed district, the DI% range was further graded into five categories *viz.*, very less infestation (VLI) (0.5–5%); less infestation (LI) (5.1–10.0%); moderately infestation (MI) (10.1–15%); high infestation (HI) (15.1–20%); and very high infestation (VHI) (≥20.1) (Table 1).

**TABLE 1:** Details of the epidemiology of FW as surveyed in pigeonpea cultivated areas under the districts of four cultivated Indian states.

Sr. no.	State	Districts	Tehsil/Village	*DI%	Infestation level
1	Andhra Pradesh	Warangal	Kamalapuram	10.66	MI
2			Ramnagar	12.33	MI
3			Shakapur	4.66	VLI
4	Madhya Pradesh	Barwani	Anjad	26.0	VHI
5			Julwania	8.5	LI
6			Rajpur	19.0	HI
7		Dhar	Dharampuri	15.6	HI
8			Kukshi	13.0	MI
9			Manawar	10.2	MI
10		Khargon	Bhikangaon	5.0	VLI
11			Maheshwar	14.5	MI
12			Mandleshwar	8.6	LI
13			Sanawad	23.8	VHI
14	Tamil Nadu	Dharmapuri	Baisalyae	6.66	LI
15			Perayambetti gate	4.50	VLI
16			Periyampatti	12.88	MI
17		Krishnagiri	Andipatti	13.25	MI
18			Bheemanak Palli	10.8	MI
19			Doddooru	5.60	LI
20	Telangana	Medak	Arur	7.24	LI
21			Ismailkhanpet	8.55	LI
22			Nandikandi	15.89	HI
23	West Bengal	Murshidabad	Nabagram	18.1	HI
24		Nadia	Karimpur	10.0	LI
25			Birohi	15.6	HI

*DI, disease incidence; 0.5–5% indicates the area under very less infestation (VLI); 5.1–10.0% indicates the area under less infestation (LI); 10.1–15% indicates the area under moderately infestation (MI); 15.1–20% indicates the area under high infestation (HI), and ≤20.1 indicates the area under very high infestation (VHI)

### Collection of Seed Materials and Plant Growth Conditions

For exploring new locally grown landraces of pigeonpea, a field survey was undertaken simultaneously with the disease survey for the collection of seeds from landraces in such areas (villages) where FW was observed naturally. Seeds from a total of six different pigeonpea landraces (Desi Nimar, Desi Tur, Grider, Parwati, Richa, and WB-20/105) were collected from different farmer’s fields during survey in different pigeonpea growing states where landraces or local cultivars are exclusively cultivated by the farmer communities year after year. Two experimental and standard pigeonpea genotypes ICP8863 and ICP2376 well-known for high resistance and high susceptibility, respectively to FW, used in this study as experimental control were procured from ICRISAT, Patancheru, India (Table 2). These landraces were not even exposed or used by the scientific communities or plant breeders to develop superior varieties or conserve their germplasm in regional or national institutes with a specific accession number. After getting their local name from the respective farmer communities, the seeds of a specific landrace were exclusively collected from a single plant selected based on growth, health, and yield. The crop was identified informally by the main author and the respective farmers, as it was well-known and a widely cultivated landrace. The apparently bold and healthy seeds of all the collected pigeonpea landraces/genotypes were surface-sterilized using 0.1% HgCl_2_, followed by washing thrice with sterile water. The treated seeds were then sown in polythene bags filled with sterilized river sand and watered properly. The polythene bags were then kept carefully in a glasshouse maintained at 28–30 ± 2°C with natural photoperiod conditions. The 7-day-old seedlings were then used for the pathogenicity test and other molecular studies.

**TABLE 2:** Passport details of collected pigeonpea seeds and their disease evaluation against FW reaction.

Sr. no.	Type	Name	Collection area	State	Number of plants died in replications*					Avg. DI (%)^a^	Disease reaction
					R1	R2	R3	R4	R5		
1	Landrace	Desi Nimar	Julwania	Madhya Pradesh	3	1	1	1	1	23.36^c^	Susceptible
2		Desi Tur	Manawar		6	4	5	4	5	80.00^ab^	Highly susceptible
3		Grider	Kukshi		Non-viable seeds						Not applicable
4		Parwati	Mandleshwar		6	5	5	6	5	89.98^a^	Highly susceptible
5		Richa	Bhikangaon		0	2	1	0	0	10.00^c^	Resistant
6		WB-20/105	Nabagram	West Bengal	1	1	4	6	6	60.02^b^	Highly susceptible
7	Genotype	ICP 8863	ICRISAT (Experimental control)	Telangana	0	0	0	1	0	3.34^c^	Resistant
8		ICP 2376	ICRISAT (Experimental control)		5	5	6	6	5	89.98^a^	Highly susceptible

*Six plants evaluated in per replication. ^a^Average disease incidence in percent; the calculated coefficient of variation (CV) = 39.419; and the critical variation (CD) of disease reaction found significant at 1 and 5% level of significance; CD at 1% = 35.530, and CD at 5% = 26.219.

### Inoculum Preparation and Pathogenicity Test for Germplasm Screening

A highly virulent strain of FU (ICFU 109) was initially inoculated on potato dextrose agar (PDA) media. After 7 days of incubation on the PDA plate, the young FU culture with profuse conidia was further inoculated in PDB media. The FU-inoculated broth culture was kept in an incubator–shaker maintained at 25°C with a continuous shaking of 120 rpm for 6–7 days. For screening of the resistance and/or tolerance levels in pigeonpea genotypes against FU under controlled conditions, the root dip screening technique was used (Pande et al., 2012). The conidial suspension of FU was diluted with sterile water to obtain a final concentration of approximately 6 × 10^5^ spores/mL. Seven-day-old seedlings of each genotype were carefully uprooted from the germination trays and washed under running water. Root tips (0.5-cm long) of each seedling were cut off and dipped in the diluted inoculum suspension for 1–2 min for entry of the pathogens. A similar procedure was followed for the experimental control, where injured seedlings were treated with sterile distilled water. The inoculated seedlings were transplanted into six-inch pots filled with sterilized soil, sand, and farmyard manure in a ratio of 1:1:1. Each genotype was transplanted into a total of 10 pots with three seedlings per pot. Five pots of each variety were considered to represent five replications for each time point (TP) of the experiment (five replications × 2 TP). The greenhouse was maintained at 25 ± 2°C with natural light and dark conditions. At two TPs, that is, 7 and 15 days after inoculation, the plants that showed the presence or absence of wilt symptoms, were selected for data recording. In order to obtain the overall disease incidence, the percent of disease incidence was calculated using the following formula:

Disease incidence (%) = (Number of disease seedlings)/(Total number of seedlings) × 100

Based on the disease incidence, genotypes were categorized as tolerant/resistant (≤10.0%); moderately tolerant/resistant (>10.0–20.0%); susceptible (>20.0–40.0%), and highly susceptible (>40%). To observe the aggregate variability in resistance or susceptibility of the collected landraces/genotypes to FW the generated data for *in vitro* greenhouse screening was analyzed by analysis of variance (ANOVA) test. The resistance or susceptibility of the landraces was analyzed by pairwise student *t*-test against ICP8863 (FW resistant). At each TP, the inoculated seedlings were removed from the soil, washed with sterile water, completely dried with tissue paper, and rapidly snap-frozen in liquid N_2_, followed by storage at –80°C.

### Molecular Characterization of Defense-Responsive Genes Against FW in Pigeonpea

#### Sequence Retrieval for Targeting Stress-Responsive Genes

The aim of the present study was to identify some important and promising genes that played a major biological role that was directly or indirectly related to defense response against biotic stresses, mainly against fungal pathogens. Twelve genes were selected, and their corresponding nucleotide sequences with their accession numbers were retrieved from NCBI database. The information on plant sources, sequence length (bp), and length of coding sequences (CDS) were collected and tabulated (Table 3). The individual gene sequence was further subjected to the sequence alignment tool BLAST, using NCBI database^1^ to obtain the best-fit ortholog sequences belonging to the *Fabaceae* crop family, where the maximum cut-off was fixed up to 80% (Altschul et al., 1997). All the identified gene sequences from the same family were retrieved and subjected to multiple alignments using the program MULTALIN to identify the conserved region (Corpet, 1988). The CDS region, which was shared by common conserved regions of the maximum number of accessions, was marked and selected to identify the longest possible Open Reading Frame (ORF). The longest stretches of ORF sequence without any intermediate stop codons were subjected to the amino acid translation using the tool ExPasy translate^2^ to obtain the putative protein sequences. The translated amino acid sequences were finally analyzed using the protein motif finder tool to understand whether the translated amino acid sequences gave the most stable target protein motif, and the Conserved Domains Database was used^3^ for this purpose. Based on the obtained functional motif of each protein, the normal and special functional behavior of the said protein was understood. Specific functions of the proteins were further validated with reported literature to confirm whether they were actively involved in response to fungal or any other biotic stress.

**TABLE 3:** Defense related gene sequences retrieved from NCBI databases for *in silico* based screening of biotic stress-responsive genes.

Sr. no.	Gene Name	Accession number	Plant Source	Sequence type	Sequence length(bp)	CDS size (bp)
1	GroES2	DQ889509	Groundnut	Partial	736	606
2	Chalcone synthase	AJ012690	Chickpea	Full Length	1375	1170
3	Isoflavone reductase	XM004512368	Chickpea	Full Length	1156	936
4	Methionine sulfoxide reductase	XM003608291	Barrel clover	Full Length	862	570
5	Glycosyltransferase	NM001249894	Soybean	Full Length	1990	1476
6	Chitinase	NM001249894	Chickpea	Full Length	1077	882
7	β-1, 3 glucanase	NM001279135	Chickpea	Full Length	1261	996
8	Osmotin	AB370233	Soybean	Full Length	909	723
9	Protease	XM_00363	Barrel clover	Full Length	1473	1473
10	Ascorbate peroxidase	FJ914865	Chickpea	Full Length	1030	840
11	Glutathione peroxidase	DQ889534	Peanut	Full Length	703	648
12	Superoxide dismutase	AJ012739	Chickpea	Full Length	702	459

#### Primer Designing and Constructing RT-PCR Profile

Oligonucleotide primers were designed for the reverse transcription-polymerase chain reaction (RT-PCR [experiment for four defense-responsive genes *viz*. the PR genes, CHS, β-1,3-glucanase, the AO genes ascorbate peroxidase (APX) and SOD]. The genes were initially screened through an *in silico* gene targeting study, as discussed earlier (section “Sequence Retrieval for Targeting Stress-Responsive Genes”). The online tool of Integrated DNA Technology^4^ was used to design the primers. Adequate quantity of high-quality total RNA from healthy and diseased pigeonpea seedlings was isolated after some optimization of a classical RNA isolation technique (Biswas and Ghosh, 2016).

The experimental ImProm-II^TM^ Reverse Transcription System (Promega, United States) was used to synthesize first-strand cDNA from the isolated RNA. For each reaction, experimental RNA (up to 1 μg) was combined with the cDNA primer [Oligo (dT) 15 @ 0.5 μg/reaction] in nuclease-free water to make a final volume of 5 μL per reaction. All the subsequent reactions of RT-PCR were strictly followed according to the manufacturer’s instructions. The recommended temperature-time profile was followed after preparing the final 20 μL RT reaction mixture and placing it in a thermal cycler. During the next step of PCR reactions, the cDNA mixture was further amplified by PCR (GenAmp, Applied Biosystems, United States) with 25 μL of total reaction volume. As the calculated Tm values for all the selected four primers ranged between 52 and 60°C, the gradient scale was adjusted for individual genes by placing five replicate reactions in different gradient blocks. After amplification, all the samples were stored at −20°C until further use. The PCR-amplified products were resolved using 1.2% agarose gel electrophoresis, visualized with UV Trans-illuminator, and photographed using the Alpha Digi DocTM system.

### Expression Profiling of FW Stress-Responsive Genes by qPCR in Pigeonpea

The cDNA was quantified using a Nanodrop spectrophotometer (NanoDrop 2000C, Thermo Fisher Scientific, Wilmington, United States), and the integrity of cDNA was checked by 1.2% agarose gel electrophoresis and ethidium bromide (EtBr) staining. For determining the expression profile of known PR and AO genes, a qPCR study was conducted. The specific oligonucleotide primers for qPCR were designed by the IDT (Integrated DNA Technology) tool (Table 4). The IF4α-Initiation component 4a gene, as the most stable housekeeping gene in pigeonpea, was considered to be an internal standard for the endogenous control (Sinha et al., 2015). The PCR reaction mixture was prepared using the following reagents: 10 μL of 2x KAPA SYBR^®^ FAST qPCR Master Mix (Wilmington, United States), 1 μL each of 10 mM PCR primers (Forward and Reverse), 5 μL of 40 ng/μL cDNA template, and 3 μL of PCR-grade water. The qPCR was carried out by completing 30 cycles using the LightCycler^®^ 480 Real-Time PCR System (Roche, United States), by following the standard cycles mentioned in the instruction manual. All the technical replications of the qRT-PCR experiments were conducted in triplicate and repeated twice to validate the robustness of the experiment. Using the quantitative values of the cDNAs, expression profiling of an individual gene was quantified at variable stress conditions by using algorithms that were truly based on the 2^–ΔΔCT^ method (Livak and Schmittgen, 2001).

**TABLE 4:** List of the selected primers used for RT-PCR based identification and qPCR-based expression analysis of biotic stress responsive genes.

Sr. no.	Gene name	Details of oligos for RT-PCR				Details of oligos for qPCR			
		Name	Sequence (5′→3′)	Length (bp)	Amplicon size (bp)	Name	Sequence (5′→3′)	Length (bp)	Amplicon size (bp)
1	Chalcone synthase	CHS-F	TTGGATGCTAGGCAAGACATGGTGGT	26	561	qCHS-F	GCTTCGTTTGGCCAAGGATTTG	22	96
		CHS-R	CTTTGAGACAATCCCAGGAACATC	24		qCHS-R	CTTGGGCCACGGAATGTGACAG	22	
2	β-1,3 glucanase	B1.3G-F	ACGGTCTCGATATCGCTGTTAG	22	453	qB13G-F	CCTACGCTAACGACCAACAA	20	129
		B1.3G-R	GTGAGTTGTCAAATATAAGAGGGTCTG	27		qB13G-R	CTTTCTCGAGAGCAGCGTATAG	22	
3	Ascorbate peroxidase	APX-F	CTGGGACTATTAACTTCAGTCAGG	24	354	qAPX-F	TAACTGGTGGACCCGAAGTA	20	121
		APX-R	CCCAATAATACCACAAGCTACTCT	24		qAPX-R	TTGCCGAACACATCCCTAAG	20	
4	Superoxide dismutase	SOD-F	TCCTAGGTGTCTCAAATGATGC	22	404	qSOD-F	GGAGCACATTTCAATCCTAATGG	23	95
		SOD-R	GGTACCCAACTTCATTATTTCCTTG	25		qSOD-R	CCGACATTGACATTCCCTAGAT	22	

### *In silico* Characterization of Stress-Responsive Genes in Correlation With Fungal Infection

Out of the four-gene transcripts amplified using qRT-PCR, two candidate genes, namely CHS and SOD, representing the PR and AO gene families, respectively, were sequenced on both sides (Xcelris Genomics, Gujarat, India). The obtained sequences were aligned and purified. The sequences of purified CHS and SOD were validated using NCBI-BLAST and deposited in GenBank. The transcript sequences of both the genes were analyzed at the *in-silico* level. Gene predictions of the CHS and SOD sequences were performed by FGENESH^5^, using the eukaryotic gene finder module (Solovyev et al., 2006). Active sites of the transcripts were predicted by the Prosite online server of ExPasy^6^ (Gasteiger et al., 2003). The motifs of the secondary protein structures were created by PDBsum using version 3.0 of Gail Hutchinson’s PROMOTIF program (Laskowski, 2001). The created motifs were then used for 3D modeling and structure prediction. As CHS and SOD exist in the homodimer state in the cell, the 3D models were predicted using homology-based modeling by the tool SWISS-MODEL^7^ using default parameters (Waterhouse et al., 2018). The 3D structures *of* predicted models were visualized using the software UCSF Chimera version 1.13.1 (Pettersen et al., 2004). The energy-minimized models were used for generation of the Ramachandran plot of the proteins using PDBsum server^8^. In order to calculate the physicochemical properties of the sequences of both CHS and SOD proteins, the software protPARAM was used^9^. The QMEAN server^10^ was used to evaluate the overall quality of the 3D structure (Benkert et al., 2011). The 3D model for ligand cellobiohydrolase-c (CBH-c) was predicted using SWISS-MODEL, and the structure analysis was carried out as mentioned above. The protein–protein docking was performed by FRODOCK version 2.0 for interactive protein–protein docking (Garzon et al., 2009). A further simulation study was carried out using the online server GRAMM-X Protein–Protein Docking Web Server version 1.2.0 by Vakser Lab^11^, to check all their possible interactions (Tovchigrechko and Vakser, 2006).

## Results

### Survey and Epidemiological Study

**FIGURE 1:**
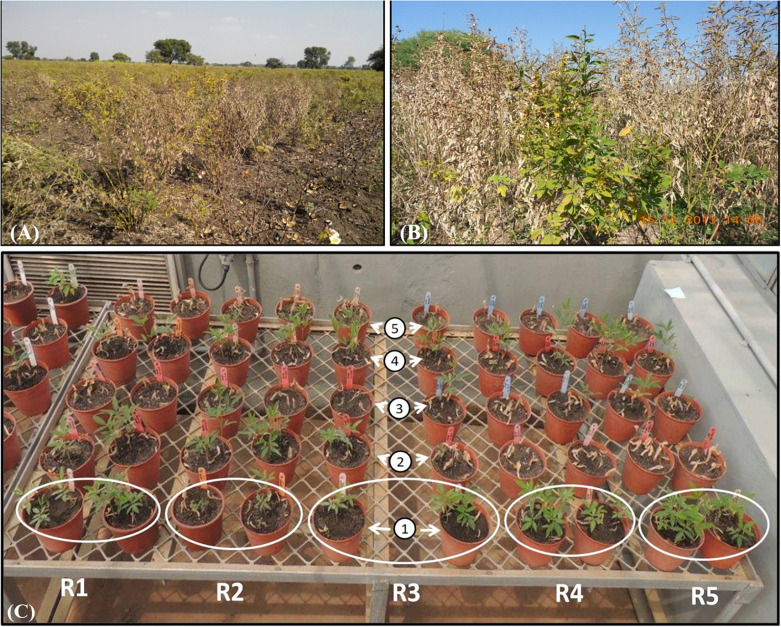
Phenotypic expression due to FWdisease in pigeonpea. Highly wilt infestation in farmer’s field at **(A)** Anjad village of district Barwani, and **(B)** Sanawad village of district Khargon from Madhya Pradesh. **(C)** *In vitro* greenhouse screening of collected five pigeonpea landraces (1: Richa, 2: WB 20/105, 3: Parwati, 4: Desi Nimar, and 5: Desi Tur) to FW; white circle indicates the replications designated by “R.”

During the extensive survey in the period between October 2014 and January 2015, 25 different villages/tehsils from four major pigeonpea growing states of India—TN, MP, AP, and WB—were covered to carry out the epidemiological studies and search for new FW-tolerant pigeonpea sources from the existing genotypes, which were indigenously grown in the surveyed areas. The maximum number of areas was covered in MP as there was no report on such a survey in this state, which contributes around 10% of the total pigeonpea production in India (Table 1). The lowest DI% (4.5%) was observed in the Perayambetti gate village of Dharmapuri district in TN, whereas the highest DI% (26.0%) was observed at Anjad village in the Barwani district of MP (Figure 1), followed by Sanawad village (23.8%) in Khargone district of the same state. As mentioned earlier, maximum coverage of the surveyed areas was done in MP, where three districts (Dhar, Khargone, and Barwani) displayed the highest level of epidemiological variation in terms of DI% (Table 1). Regarding the overall disease incidence, all four states showed significant variations in terms of disease epidemiology. The seed samples of pigeonpea were exclusively collected from the areas where local landraces of pigeonpea are cultivated mostly and having lower FW disease incidence. Out of six landraces, the five landraces were collected from different villages of MP having low incidence of FW. For example, the seeds of landraces Richa and Parwati were collected from Bhikangaon and Mandleshwar village of Khargon district where DI were 5.0 and 8.6%, respectively. Similarly, the seeds of Desi Tur and Grider were collected from Manawar and Kukshi village of Dhar district where DI were 10.2 and 13.0%. Furthermore, the seeds of Desi Nimar was collected from Julwania village of Barwani district where the wilt DI was 8.5%. The only pigeonpea landrace WB-20/105 was collected from Nabagram village of Murshidabad district of WB where FW-DI was 10.0%. The pigeonpea seeds, representing a total of eight unique genotypes, were collected from three different states: MP, AP, and WB (Table 2). The seed has been collected from such surveyed areas where the DI% are comparatively lower (Give Range).

### Exposure to Stress and Screening for Tolerance

Out of seven genotypes/landraces used for resistance screening (excluding Gridar as non-viable seeds), only two, ICP8863 and Richa, were found to be promising, with the least incidence of wilt. In case of ICP8863, that is known to be resistant to FW, a DI% of only 3.33% was recorded. The DI% in Richa was 10%, thus placing this landrace into the highly resistant category. On the other hand, another landrace “Desi Nimar” was categorized as moderately tolerant, with a DI% of 23.3%. All the remaining three landraces, Parwati, Desi Tur, WB–20/105, and ICP2376, were found to be highly susceptible to FW (Table 2). In ANOVA analysis, the landraces were found to be significantly varied in their susceptibility and resistance to FW at 1% (CD = 35.530) as well as 5% (CD = 26.219) level (Table 2 and Supplementary Table S1). In pairwise “t” test, it was found that disease incidence there was no significant difference between landrace Richa and genotype ICP 8863 in resistance level (Supplementary Table S2).

### Identification and Molecular Characterization of Biotic Stress-Responsive Genes in Pigeonpea

#### *In silico* Study

For the *in silico* analysis of biotic stress-responsive genes, a total of 12 diversified genes that were directly or indirectly involved in defense response in plants were selected, with some falling under a specific class of functional genes. Prior to the multiple sequence alignment, all the selected sequences of a single gene were graphically aligned to understand the level of divergence and convergence within ortholog sequences. In the case of CHS, the CDS region covered 58–1227 nucleotides, with a length of 1,170 bases covering the complete CDS sequence. Similarly, full-length CDS coverage was observed in maximum orthologs of a given gene, particularly in the case of isoflavone reductase (IFR), methionine sulfoxide reductase B2 (MSR), glycosyltransferase (GTF), chitinase, β-1,3-glucanase, and osmotin genes. In another case, genes encoding protease and APX had the full-length CDS for a single gene sequence, but the major orthologs did not cover this region of the target gene, due to their shorter size (Table 3). The gene glutathione peroxidase (GPX) was observed to have the lowest match in the CDS region (120 ntd.), from a full-length CDS of 648 bases. Furthermore, the gene SOD consisted of the lowest CDS (649 ntd.), but only conserved 500 bases (1–500 ntd.) in the ortholog sequence. Apart from the graphical alignments of all the gene accessions, multiple alignments were also performed using all the mentioned orthologs of each gene, to revalidate the conservation in the CDS region (Thompson et al., 1994). For the above-mentioned 12 gene sequences, the maximum conservation in terms of length was visually observed in the case of nine genes, except osmotin, protease, and IFR. The phylogenetic tree was constructed for the selected nine gene sequences by considering only the full-length CDS region of each gene and partial CDS when the full-length sequence was not available. From the cladogram analysis of all the selected phylogenetic trees, the maximum number of accessions grouped with a target sequence in a single cluster was observed in the case of APX and SOD (AO enzyme family), and CHS and β–1,3-glucanase (PR protein family), with a defined biological and molecular defense response against biotic and abiotic stresses (Padaria et al., 2014). Therefore, these four genes were finally selected for further molecular experiments.

#### Reverse Transcription-Polymerase Chain Reaction (RT-PCR)

After several optimizations of the gradient scale in PCR for each transcript, the best annealing temperatures that were observed to be reproducible for successful amplification were 52.6°C for *CHS*, 60.0°C for *APX*, and 57.0°C for β*-1,3-glucanase* and *SOD*. The desired amplicons of the expected size (*CHS* ≈ 560 bp, *SOD* ≈ 400 bp, *APX* ≈ 350 bp, and β*-1,3-glucanase* ≈ 450 bp) were obtained, followed by analyses on 1.0% agarose/EtBr gel to ensure the successful molecular identification of these four defense-responsive genes (Supplementary Figure S1). The identification of these four genes was performed up to the RT-PCR level, as all the amplicons were derived from cDNA rather than genomic DNA, showing that these were specific, amplified, and unique bands of the desired length. Therefore, our study ensured the presence of gene transcripts at the mRNA level, and it was sufficient to authenticate their expression pattern by using the real-time PCR approach.

### Validation of FW Stress-Responsive Genes by qRT-PCR in Pigeonpea

**FIGURE 2:**
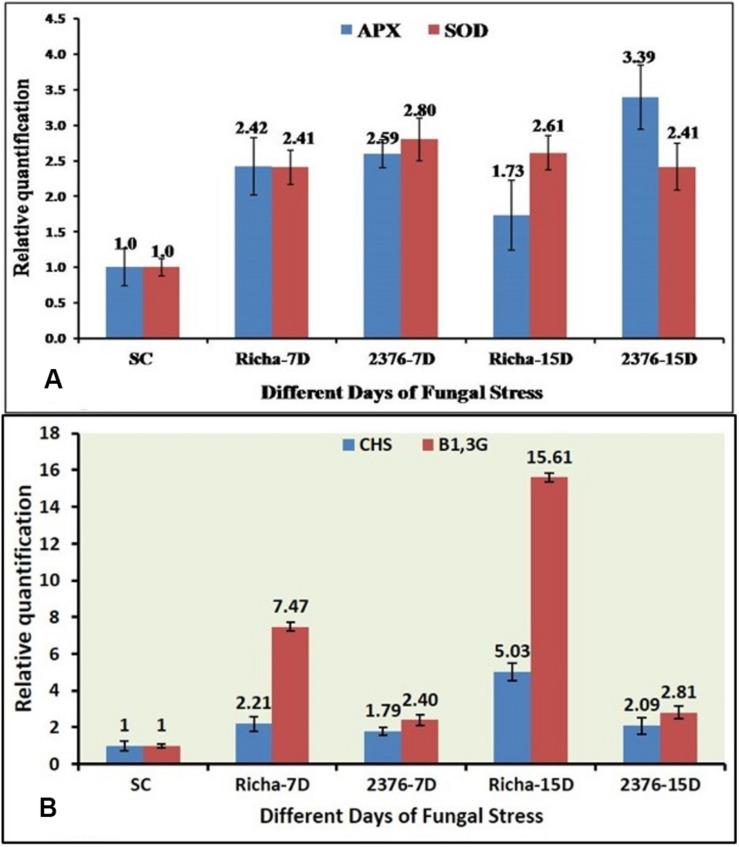
qPCR-based comparative expression pattern analysis of **(A)** two antioxidant genes (APX & SOD), **(B)** two PR genes (CHS & β-1,3-Glu) under FU infection in two pigeonpea genotypes; SC-ICP2376 as Susceptible Control, 7D – 7 days after inoculation, 15D – 15 days after inoculation, Richa – Genotype of unknown response, 2376 – A susceptible variety.

In this study, the expression profiling of four defense-related genes in susceptible genotype ICP2376 and resistant (Richa) landrace of pigeonpea, during the development of FW, was determined through qPCR. In the comparative analysis of AO gene expression in pigeonpea during FU infection, it was observed that both *APX* and *SOD* were upregulated in inoculated plants, irrespective of the genotype (Tarafdar et al., 2018). Overall, the expression of the *SOD* gene was observed to be relatively higher than the *APX* gene in the later stage of infection in the susceptible cultivar ICP2376. Although the expression levels varied between different TPs at 7DAI and 15DAI, the expression was differentially regulated by a combination of positive (ICP2376) and negative induction (Richa) (Figure 2A). It could be proposed that the degree of AO activity needs to be enhanced with the time of infection, and delayed to balance the hypersensitivity response in case of pigeonpea genotypes susceptible to FU. In the case of Richa, the difference in induction of the *APX* gene (FC −2.42 to 1.73) in two different TP could be due to the activation of anti-oxidative response immediately after the fungal attack, and this decreased gradually as other defense mechanisms of that genotype restricted the fungus. After immediate enzymatic activation, products of the *SOD* gene might have accumulated in sufficient amounts to necessitate the switching off of the gene. The effect of stress due to FW pathogenicity on *SOD* gene expression in both genotypes was quite interesting, wherein a variable range of positive induction with a gradual increase with inoculation time was observed in case of Richa, while ICP2376 conversely showed a slight decrease from 2.80 to 2.41 as TP extended up to 15 days (Figure 2A). Thus, it was confirmed that although both genes of the same functional family were induced immediately after the fungal attack, the level of expression needed to increase as the time of infection progressed to sustain the susceptible genotype with maximum defense response, whereas the resistant one could recover from an infection after some time and the *APX* gene returned to its native state.

While comparing the expression pattern between *CHS* and β*-1, 3-glucanase* genes, it was observed that they had a mixed response in terms of induction in both, the susceptible and the new genotype, with unknown H functions (Tarafdar et al., 2018). Initially, with an increase in the inoculation time, the *CHS* gene in Richa increased its expression by up to 5.03-fold from an initial level of 2.21-fold, compared to the nearly baseline expression in ICP2376 (FC 1.79–2.09). This confirmed that the said enzyme got involved in stimulating the initiation of defense response at the ground level (Figure 2B). On the other hand, as the inoculation time increased from 7DAI to 15DAI, the relative expression of β*-1, 3-glucanase* gene in ICP2376 remained more or less constant, with an apparent fold change (FC) of 2.40–2.89 (Figure 2B). In the case of Richa, a continuous change in the inoculation time further accelerated gene activity by gaining induction by up to 15.61-fold from a value of 7.47. The overall expression pattern of this gene in both genotypes showed a zigzag-type model and could possibly be explained by the fact that most gene expression is dynamic in nature and is not expected to be upregulated at all TPs of the infection.

### *In silico* Analysis and Protein Modeling

**FIGURE 3:**
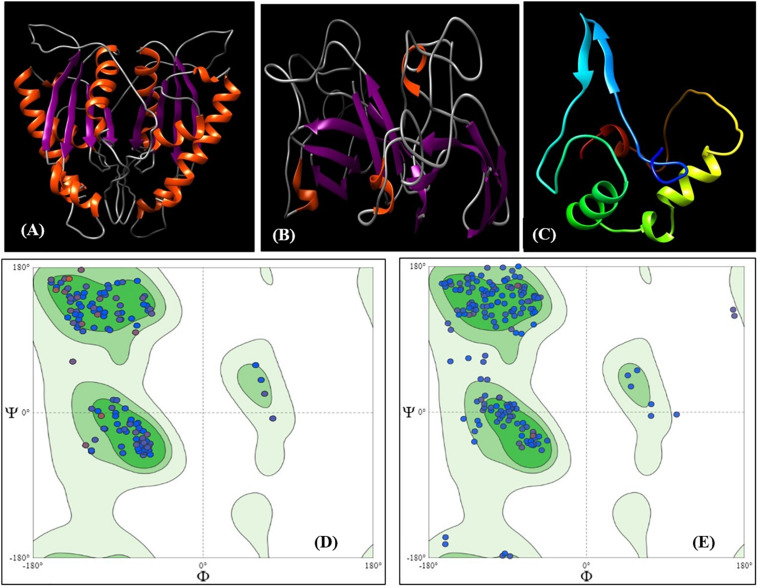
3D Model of protein CHS **(A)** and SOD **(B)** represented as ribbon models predicted using SWISSMODEL. Two similar chains represent the homodimer state of the proteins in the cell. The orange color represents β helix and purple color represents the strands. **(C)** 3D Model of ligand CBH-c with color representing residue composition. Ramachandran plot for 3D structure for **(D)** CHS and **(E)** SOD.

**FIGURE 4:**
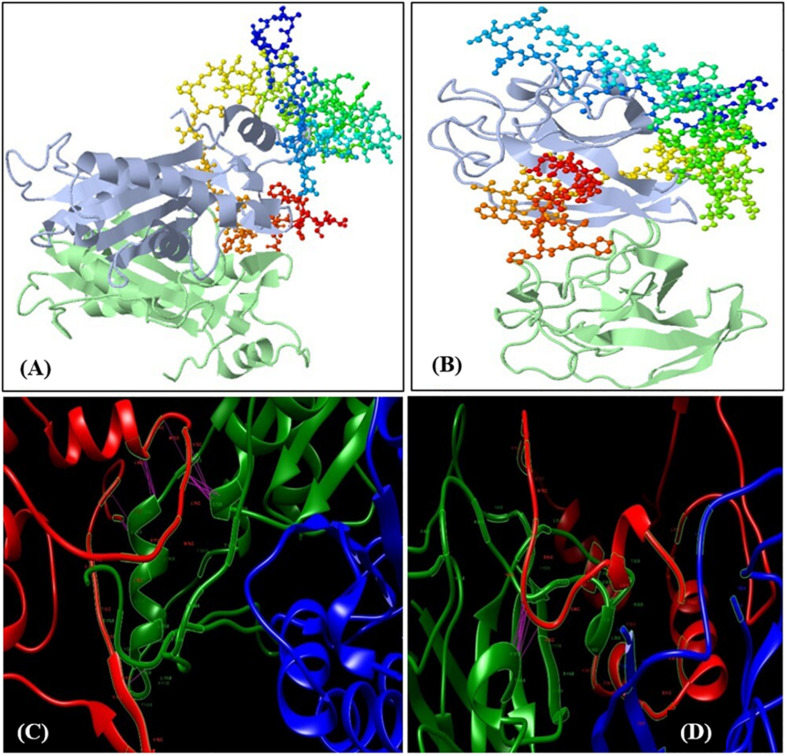
Docking of the predicted 3D models. **(A)** Docking of CBH-C with chalcone synthase (CHS) and **(B)** Docking of CBH-c with SOD. The receptors (CHS and SOD) are represented as ribbon and ligand (CBH-c) as colored balls and sticks based on residues. Lower panel shows docking simulation studies with the clashes and contact between the receptor and ligand molecules. The pink line represents the contact. Red ribbon structure is CBH-c, whereas green and blue ribbons represent the homodimer chains of CHS **(C)** and SOD **(D)**.

To validate the transcript abundance in the *Fusarium*-inoculated pigeonpea landrace Richa, the PCR-purified products of partially amplified *CHS* (537 bp) and *SOD* (361 bp) genes were sequenced. The sequences were purified and submitted to GenBank under accession numbers MN095237 and MN095238. The partial gene sequences of *CHS* and *SOD* were translated to 179 and 120 amino acids (aa), respectively. The nucleotide sequences were compared with those from other pigeonpea varieties and legumes. The sequence comparison showed 98.32% similarity for *CHS* from *Phaseolus vulgaris* [GenBank: X06411] and 97.51% similarity for SOD from *Cajanus cajan* [GenBank: XM_020353639]. The amino acid sequences deduced from both *CHS* and *SOD* had predicted molecular weights of 19.26 kDa and 12.15 kDa, respectively. The target receptor proteins from full-length CHS and SOD genes had lengths of 179 and 120 aa, respectively. Chalcone and stilbene synthase (Chalcone_synth; position 62–78) is identified as the active site in CHS, with Cys164, an active residue, at position 70. On the other hand, SOD has two active sites of copper/zinc SOD signature 1 and 2 at positions 15–25 and 109–120, respectively. Protein sequences were analyzed for the prediction of 2D and 3D models, based on homology modeling (Padaria et al., 2013; Kumar et al., 2017). The 3D model selected for this study had a similarity of 94.41% and 80% for CHS and SOD, respectively, which is much higher than the cut-off value of 30%. The structure of CHS has 54 alpha helices and three beta sheets, along with two beta hairpin loops, while SOD is composed of four alpha helices, two beta sheets, and two beta hairpin loops (Supplementary Figure S2). The 3D models for both the proteins are present in the homodimer state (Figures 3A,B). The stability of the structures can be confirmed by the presence of beta sheets in the secondary structure (Supplementary Figure S2). Based on the Physico-chemical properties calculated using the protPARAM server, the amino acid composition (Supplementary Table S3) was analyzed, and both the proteins were classified as stable proteins based on their instability index (CHS: 28.02 and SOD: 5.66) (Padaria et al., 2013). SOD is considered to be a highly stable structure due to its low instability index. Furthermore, analysis of the Ramachandran plots indicated phi (φ)/psi (ψ) angles of the amino acid residues as 96.89% and 95.19%, for CHS and SOD, respectively (Figures 3D,E), thereby confirming the stability of both the proteins. The QMEAN value was analyzed for the quality of the 3D structure. The quality scores for CHS and SOD were 0.84 and 0.85, respectively. A reliable 3D structure lies within the range 0.5–1. The 3D model of CBH-c was predicted using SWISMODEL (Figure 3C). The stability of the structure of fungal CBH-c was also checked. The instability score of 39.12 and quality score of 0.61 confirms its reliability (Kumar et al., 2017). The docking was done using FRODOCK (Figures 4A,B), and simulation was carried out using the online server GRAMM-X. Clash/contact scores were calculated for both the interaction models (Figures 4C,D). The maximum correlation score was obtained for CHS (189394.84), while for SOD, it was 178941.59. CHS docking with CBH-c had a clash score of 20 and 138 contacts, whereas SOD interacted with CBH-c with 20 clashes and 179 contacts. CBH-c bound more strongly with CHS as compared to SOD. In case of the attack by FU, the CBH gene present in the fungal cell could be considered to be a potent factor for the synthesis of cellobiohydrolase, which is secreted by fungi to degrade cellulose of the host plant through the discharge of cellobiose, for carrying out their pathogenic activities (Hatsch et al., 2004). The present interaction study of both plant genes with the FU-CBH gene could be correlated with the qPCR analysis, where CHS was observed to show a higher rate in fold change expression (7.47 at 7DAI to 15.61 at 15DAI) than SOD (2.41 at 7DAI to 2.61 at 15DAI). This indicated that the CHS gene transcript was more active than SOD, with a tendency to release a higher number of functional proteins through the process of translation after pathogen inoculation and could also inhibit the activity of the CBH protein by strongly binding to it.

## Discussion

FW is a vital ailment of pigeonpea and is recognized to be the worst hindrance in the cultivation of this crop in India (Singh, 2009). The annual economic losses due to FW in pigeonpea have been assessed at USD 36 million in India, and the subsequent loss in yield can also be increased up to 100% in susceptible cultivars (Kannaiyan et al., 1984). Therefore, it is necessary to develop a sustainable solution to prevent the disease in this crop. Among the different control measures, early diagnosis and control facilitated by the advancement in molecular technologies appear quite promising. The importance of extensive survey is related to the epidemiology of disease not only to understand, describe, compare, and predict an epidemic, but also to create a chance to explore the most promising highly tolerant genotype/s against a particular disease from the surveyed areas. The disease incidence/severity in plants is a complex phenomenon, completely based on the virulence of the pathogens (variant/races), the inert defense mechanism within the plants (resistance or susceptibility) to diseases, and variable climatic conditions (climatic elements). In this study, the surveys were carried out in different agro-climatic zones of India known for the cultivation of locally popular pigeonpea cultivars. Besides that, the different FU were reported to be present in the different agro-climatic zones of India. So, we can assume that all the reasons were involved in a varied degree of FW incidence in the different surveyed areas on pigeonpea wilt disease.

The present survey-based study has reported the regional diversity of more than 25 pigeonpea-cultivating areas, representing the major agro-climatic regions of India under pigeonpea production, those were found to be suitable for growing landraces due to their tolerance or susceptibility to FW. Considering the most variable geographical region, MP represented a great diversity in the range of disease incidence, from resistant to highly susceptible areas. As far as lowest disease epidemiology was concerned, TN was found to be the lowest disease-prone state, with no area being classified as a highly infested or very highly infested one. Apart from India, this disease has been reported from East Africa and Malawi, where yield losses have been reported to exceed 50% in some places. Yield losses in pigeonpea due to FW have also been reported from countries such as Bangladesh, Indonesia, Grenada, Myanmar, Mauritius, Nevis, Nepal, Venezuela, Trinidad and Tobago (Reddy et al., 2012). This pathogen was also reported to cause severe disease in the Southern Zambezia province in South Africa (Gwata et al., 2006).

The search for pigeonpea landraces resistant to FW has yielded an overall contrasting response for all selected genotypes. The experimental reference variety ICP8863 displayed negligible or nearly zero symptoms (DI = 3.33%) in one out of 30 plants, as it is already known for its best resistance against FW, irrespective of environmental or geographical conditions. Richa was also observed to be among the most highly tolerant landraces (DI = 6.67) after ICP8863, with a higher degree of tolerance potential, which could be due to certain morphological modifications in the xylem of the root system, targeted activation of certain defense-related proteins, and transcription factors during fungal stress, or both. On the other hand, Parwati was identified as one of the most susceptible among all the experimental landraces, which could be attributed to its weak defense system. ICP8863 and ICP2376 were identified for their extreme resistance and susceptibility, respectively, in our experiment, and have been validated several times previously as well (Chavan et al., 1995; Singh, 2009).

Therefore, the three genotypes viz. ICP8863, ICP2376, and Richa were selected for further molecular biology studies. In the overall evaluation data, all the four classes of disease scales were fitted for the selected landraces. Several efforts have been taken over time to identify the genotypes or cultivars that are most resistant against FU and other pathogenic strains. The first observations on wilt resistance in pigeonpea were reported by Butler in 1908 (Butler, 1908). Among other studies, one reported zero resistance to FW in 950 genotypes screened, with <10% wilt incidence in 19 genotypes (Kannaiyan et al., 1984); another demonstrated resistance to wilt in 16 out of 31 pigeonpea cultivars screened, with the highest degree of resistance (DI = 2.15%) in the BWR369 cultivar (Dhar et al., 2005); a study by Srivastava et al. (2018) reported resistance to FW in six cultivars out of 216 late-maturing pigeonpea germplasm evaluated; and a 2-year study at Patancheru, India, on resistance screening in 976 genotypes, germplasm, and breeding lines using the wilt sick plot, reported resistance in 92 genotypes (Karimi et al., 2012). Another study on the evaluation of new elite pigeonpea germplasm using wilt sick plots demonstrated a consistently high level of resistance (DI < 20.0%) to FW in ICEAP00040 genotype in Kenya, Malawi, and Tanzania (Gwata et al., 2006).

**FIGURE 5:**
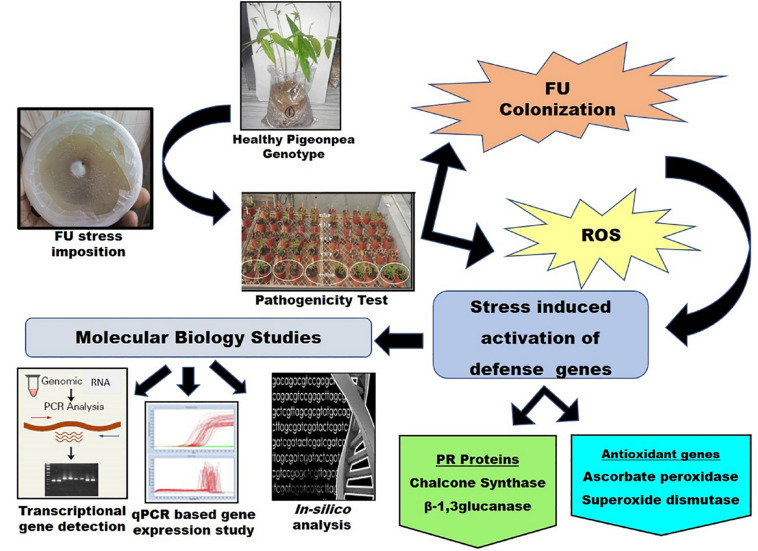
A schematic representation of the linking pathways between the present study and molecular pathogenesis of FU concomitant with ROS regeneration and PR protein induction.

For the identification, quantification, and expression profiling of highly active plant defense-responsive genes, enzymes, and in most cases, transcription factors and high-throughput molecular techniques have been developed and customized regularly for their successful application. In this study, understanding the spatio-temporal expression of major defense-responsive promising genes during pathogenesis, and the study of different patterns of symptom-based tolerance in various plant genotypes specific to different agro-climatic regions of India, can provide vital information on crop improvement with a well-developed tolerance mechanism (Figure 5). Higher plants directly defend against a number of biotic and abiotic stresses with their broad range of defense mechanisms, especially the production and accumulation of PR proteins in response to invading pathogens (Ebrahim et al., 2011; Butt et al., 2019). A wheat β–1, 3-glucanase gene (TaGlu) induced by *Puccinia striiformis* (Pst) was cloned and characterized, and the qPCR analysis confirmed that the transcription of TaGlu was induced in both compatible and incompatible interactions (Liu et al., 2010). In this study, the modified RNA extraction method was equally useful to extract RNA from the stressed sample as well as the fungus with maximum reproducibility, to trace the functions and behavior of the genes under various stresses during pathogen attack (Biswas and Ghosh, 2016). Using RNA isolated by a modified method, RT-PCR-based detection of four genes belonging to AO enzyme and PR protein groups was performed, and their amplification indicated that the genes are actively present in the plant system during FW stress. The corresponding mRNAs were synthesized in the nucleus and circulated to the cytoplasm, followed by their translation to the corresponding proteins or enzymes, which were directly or indirectly involved in the defense response to significantly enhance the plant’s tolerance to pathogen attack. To precisely monitor the expression levels of genes at various stages, qPCR-based transcript profiling approach is highly appealing, and the present comparative analysis of the expression of AO genes with PR protein genes has revealed a variable mode of expression between susceptible and biologically validated resistant cultivars.

It was also observed that there was no significant difference exist in the induction of *SOD* for Richa and ICP2376 and in case of *CHS* for ICP2376. This suggests that these genes did not have any significant variation in expression under FW stress. A higher level of transcript expression at the last TP confirms that the expression was specific to the stage of infection, rather than the gene or genotype. This may help the chickpea plant to cope with the deleterious effects of pathogen attack at an early stage, which is critical for its subsequent growth and development (Gutiérrez et al., 2012). Comparatively, significant variations were observed in the case of *APX* and β-1, 3-glucanase genes. A major finding of the present study was the differential expression of *CHS* gene in susceptible and resistant (biologically validated) pigeonpea genotypes, whereas the level of induction of β-1, 3-glucanase was observed to increase in the Richa under continuous pathogen load. Liu et al. (2010) and Nagy et al. (2004) also reported a similar pattern of expression of the β-1, 3-glucanase gene induced by the stripe rust pathogen *Puccinia striiformis* f. sp. *Tritici* in wheat, and *CHS* gene induced in response to pathogen infection in Norway spruce phloem, respectively. It is known that plants possess inducible and constitutive defenses. Structural analysis of the PR and AO genes suggests that they act in signaling cascade(s) that coordinate the initial plant defense responses in order to inhibit the growth of pathogens. Besides, products of PR genes may facilitate plant development, and therefore be expressed in the challenging but tolerant genotypes of pigeonpea plants, ready to detect any attack. Our results suggest that both mechanisms may be important for tolerance to FU, although the difference between tolerance and susceptibility depends on a number of internal and external factors, among which, the early detection of pathogen is considered to be a crucial factor.

The *in silico* 3D structure for the proteins CHS and SOD was predicted in this study. The value of instability index (CHS: 28.02 and SOD: 5.66) indicated their high stability as a model. The GMQE score of CHS (0.99) and SOD (0.93) indicated the reliability of the model from target template alignment and target sequence coverage. The determined quality values of 0.84 and 0.85 for CHS and SOD, respectively, were within the acceptable range for a high-quality model. Further validation and refinements of the model were carried out using the Ramachandran plot. The phi (φ)/psi(ψ) angles of the amino acid residues revealed that both CHS and SOD proteins held more than 90% amino acids in the favored region, thus indicating the model’s accuracy. The identified active site in CHS (Cys164) has been reported to be crucial in the formation of polyketides and initiation of the series of decarboxylation, condensation, and cyclization reactions, along with other amino acids (Tropf et al., 1995; Dao et al., 2011). SOD shields cells from ROS by catalyzing the disproportionation of superoxide anion radicals into molecular oxygen and hydrogen peroxide. Predicted active site copper/zinc SOD signature 1 and 2 at regions 15–25 and 109–120 contributes to the stability of the framework and dimer assembly (Perry et al., 2010). The maximum correlation between docking score was considered to be the strongest protein-ligand interaction. Thus, both CHS and SOD actively bind to CBH-c in order to block the functional activity.

In conclusion, evaluation of the survey results revealed the feasibility of pre-screening of genotypes falling under the resistant group (D.I. 0–10%) for genetic diversity analysis and their potential association with FW tolerance. A summarized representation of the linking pathways in the present study and the molecular pathogenesis of FU concomitant with ROS regeneration and PR protein induction is presented in Figure 5. Based on our method, two well-known pigeonpea varieties (ICP8863 and ICP2376) showed similar sensitivity to FU, as reported earlier. Therefore, the current survey and resistance screening results could be considered to be adequate in identifying Richa as the best performing highly tolerant landrace to FW in this study. The aim of the molecular study was to identify promising genes and robust alleles for fungal disease resistance, which could be further utilized for the development of crops resisting FW infection. Therefore, we performed the molecular detection of four defense-related genes through an RT-PCR-based approach and thereby quantified their expression level using qPCR technology. The β-1, 3-glucanase under PR protein family was highly functional, and the *CHS* gene under polyketide synthase super family was moderately functional in the tolerant genotype. In another case, *APX* and *SOD* under the AO group were also upregulated to trigger and modulate the resistance response, with low variation between the resistant and sensitive genotypes. Such genes could be potentially useful molecular markers for screening resistance genes from pigeonpea and could be used for developing transgenic and/or improved pigeonpea varieties resistant to FW infection, suitable for the changing global climate.

## Data Availability Statement

The datasets presented in this study can be found in online repositories. The names of the repository/repositories and accession number(s) can be found in the article/Supplementary Material.

## Author Contributions

KB, PG, and MS conceptualized the idea and designed the experimentation methodology. KB, AT, RK, and NS contributed in data curation and analysis of data, original draft preparation, visualization, and investigation. PS and SP contributed significantly in reviewing and final editing of the manuscript. All authors read and approved the manuscript.

## Conflict of Interest

The authors declare that the research was conducted in the absence of any commercial or financial relationships that could be construed as a potential conflict of interest.

## Footnotes

1 http://www.ncbi.nlm.nih.gov/blast

2 http://web.expasy.org/translate/

3 http://www.ncbi.nlm.nih.gov/cdd/

4 http://eu.idtdna.com/primerquest/home/index

5 http://linux1.softberry.com/

6 https://prosite.expasy.org/

7 https://swissmodel.expasy.org/

8 http://www.ebi.ac.uk/thornton-srv/databases/pdbsum

9 http://web.expasy.org/protparam/

10 https://swissmodel.expasy.org/qmean/

11 http://vakser.compbio.ku.edu/resources/gramm/grammx/index_html
