# Supplementary material for: Molecular Analysis of Disease-Responsive Genes Revealing the Resistance Potential Against Fusarium Wilt (Fusarium udum Butler) Dependent on Genotype Variability in the Leguminous Crop Pigeonpea
Source: Front Genet. 2020 Aug 20;11:862. doi: 10.3389/fgene.2020.00862 (PMC7468401; doi:10.3389/fgene.2020.00862)
Supplement: Supplementary file 1 [file Data_Sheet_1.pdf]

Supplementary Figures

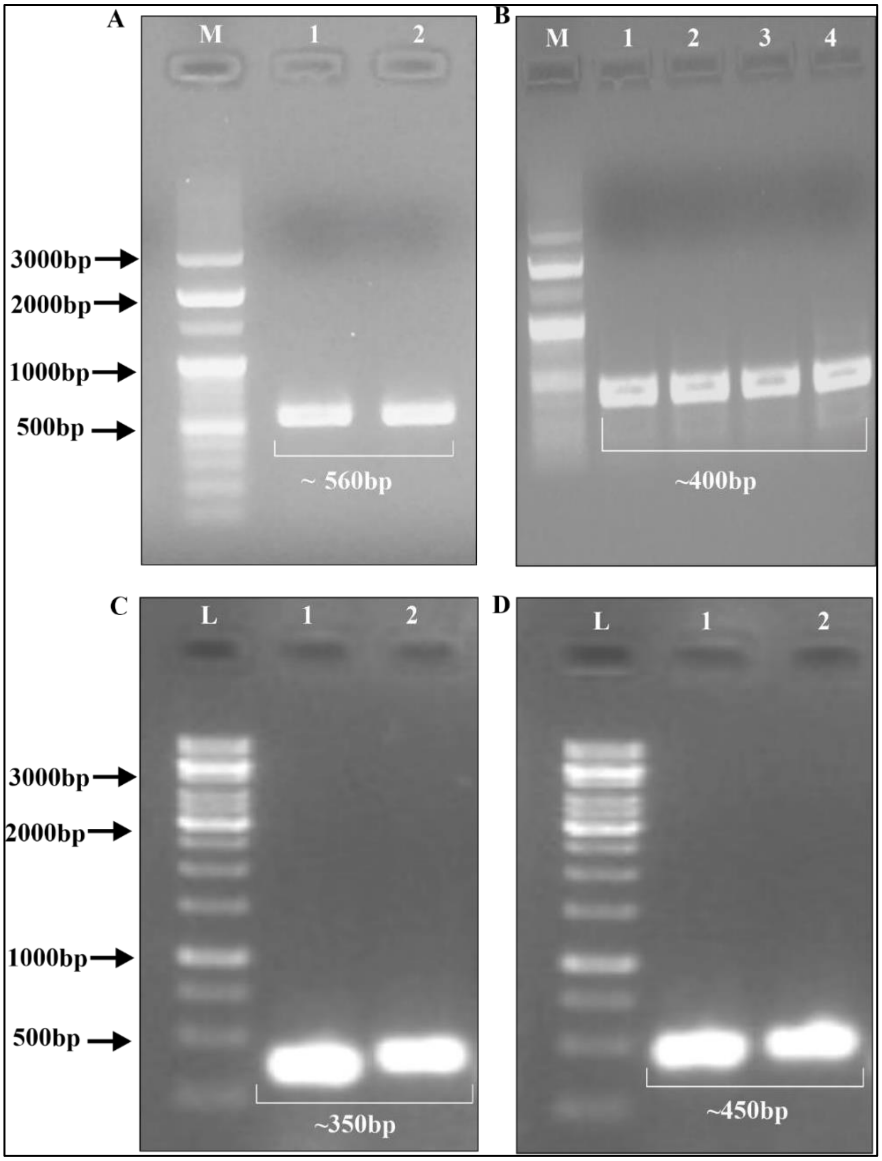

Figure S1. Agarose gel electrophoresis of PCR derived amplicons of four defense related genes from fungal inoculated Richa variety. (A) Lane 1 and 2 depicting that CHS gene sharply amplified (560bp) at 52.6 ^{o}C and 53.4 ^{o}C gradient temperatures; (B) SOD gene amplification (400bp) clearly visible in four lanes at 56.8 ^{o}C, 57.2 ^{o}C, 57.6 ^{o}C and 57.9 ^{o}C gradient temperatures; (C) APX gene amplicons of 350 bp in lane 1- Richa and lane 2- ICP8863 (D) β- 1,3-glucanases gene amplicons of 450 bp in lane 1- Richa and lane 2- ICP8863; M-1kb DNA ladder, L-100bp DNA ladder.

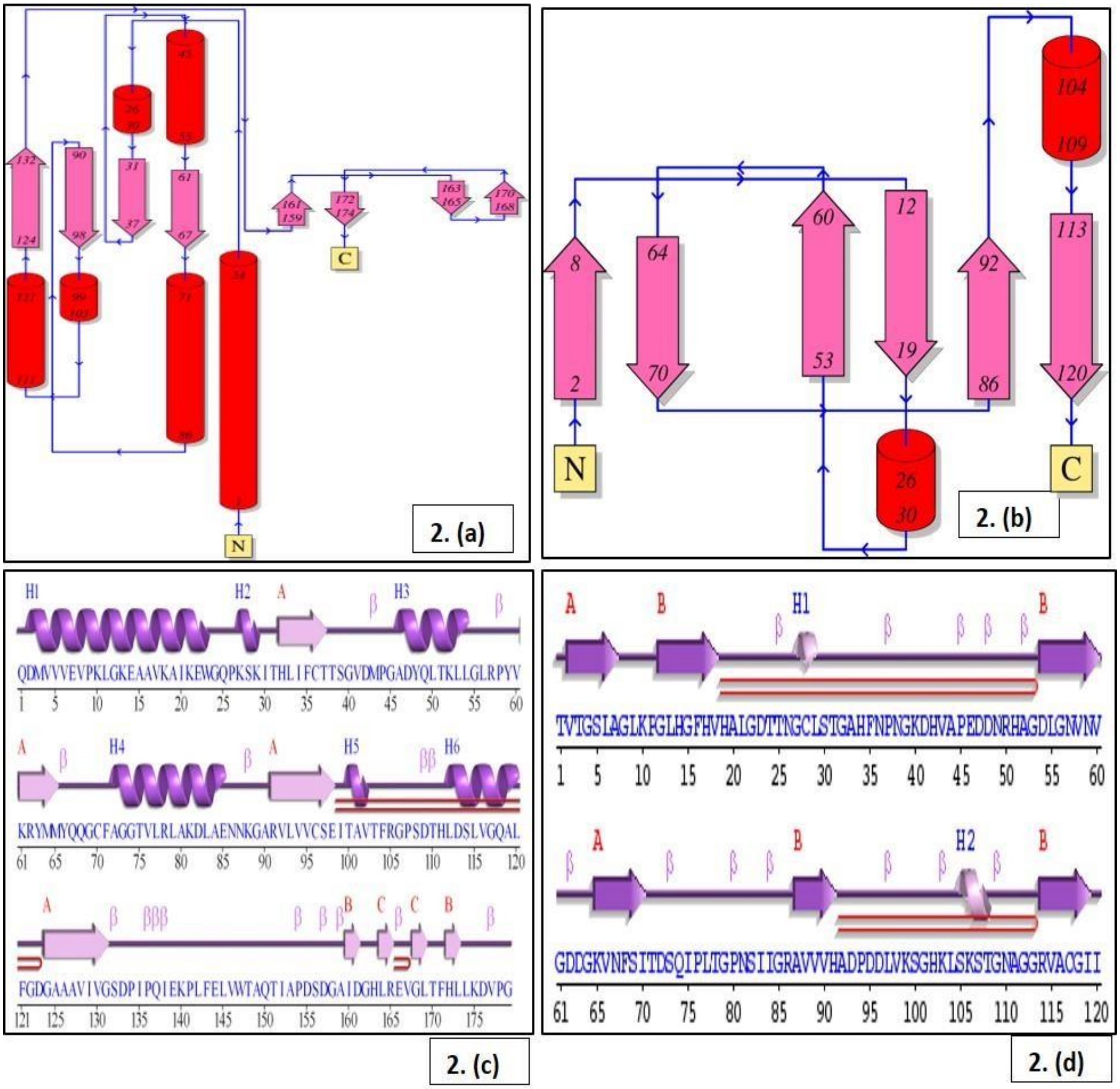

Figure S2. Topology of CHS (a) and SOD (b). Predicted secondary structure of CHS (c) and SOD (d); the structure consist of helics (H) and strands by the sheet A, B, C and motif having β helix turn and β hairpin loops.

Supplementary Tables

Table S1: ANOVA test landraces/genotypes for their resistance or susceptibility to Fusarium wilt.

Source of variation	Degrees of freedom	Sum of squares	Mean sum of squares	F cal	F prob
**Replications**	4	762.019	190.505	0.472	0.756
**Genotypes**	6	43410.264	7235.044	17.935	0.000
**Error**	24	9681.737	403.406	-	-
**Total**	34	-	-	-	-

Table S2: Statistical comparison of collected pigeonpea landraces with genotype ICP 8863 for their resistance or susceptibility to Fusarium wilt.

Landrace name	Variance	T-Statistics
Richa	222.194	1.5**
Desi Nimar	222.178	3.501*
Parwati	83.367	22.041
Desi Tur	194.389	12.83
WB-20/105	1749.867	3.207*

T-Table values are 2.776 and 4.604 at 0.05% and 0.01%, respectively

** Not significantly different at 1 and 5% level;

* Significantly different at 5% level

Table S3. Physiochemica[illegible]operties of the protein CHS and SOD showing the amino acid composition.

Amino acid		CHS		SOD		CBH-[illegible]	
Ala	(A)	36	9.30%	11	7.20%	5	5.40%
Arg	(R)	17	4.40%	3	2.00%	2	2.20%
Asn	(N)	13	3.30%	10	6.60%	6	6.50%
Asp	(D)	21	5.40%	10	6.60%	7	7.60%
Cys	(C)	7	1.80%	2	1.30%	1	1.10%
Gln	(Q)	14	3.60%	3	2.00%	2	2.20%
Glu	(E)	24	6.20%	5	3.30%	2	2.20%
Gly	(G)	30	7.70%	29	19.10%	9	9.80%
His	(H)	7	1.80%	8	5.30%	4	4.30%
Ile	(I)	23	5.90%	7	4.60%	3	3.30%
Leu	(L)	37	9.50%	11	7.20%	8	8.70%
Lys	(K)	26	6.70%	6	3.90%	7	7.60%
Met	(M)	14	3.60%	1	0.70%	4	4.30%
Phe	(F)	14	3.60%	5	3.30%	3	3.30%
Pro	(P)	21	5.40%	7	4.60%	2	2.20%
Ser	(S)	20	5.10%	10	6.60%	13	14.10%
Thr	(T)	21	5.40%	11	7.20%	4	4.30%
Trp	(W)	4	1.00%	0	0.00%	3	3.30%
Tyr	(Y)	12	3.10%	0	0.00%	1	1.10%
Val	(V)	28	7.20%	13	8.60%	6	6.50%
Pyl	(O)	0	0.00%	0	0.00%	0	0.00%
Sec	(U)	0	0.00%	0	0.00%	0	0.00%
